# Study on differences in flavonoid synthesis in *Xanthoceras sorbifolia* leaves based on transcriptome analysis

**DOI:** 10.3389/fpls.2026.1822700

**Published:** 2026-06-19

**Authors:** Baotong Zheng, Xiaojiao Zhou, Xing Tao, Ya Qi, Yao Wei, Zhibiao Xu, Gaiping Wang

**Affiliations:** 1 Nanjing Forestry University, College of Soil and Water Conservation, College of Forestry and Grassland, Collaborative Innovation Center for Sustainable Forestry in Southern China, Nanjing, China; 2 Yancheng Forest Farm, Yancheng, China

**Keywords:** *4CL*, *C12RT1*, flavonoids, *LAR*, leaves, transcriptomic, *Xanthoceras sorbifolia*

## Abstract

**Introduction:**

*Xanthoceras sorbifolia*, an oil-rich woody plant, shows significant potential for biodiesel production from its seeds. Its leaves contain flavonoids with medicinal value, yet the underlying molecular mechanisms of flavonoid biosynthesis remain elusive.

**Methods:**

In this study, we collected mature leaves from the upper and middle parts of three *Xanthoceras sorbifolia* clone plants exhibiting significantly different levels of flavonoids and performed transcriptome analysis.

**Results:**

The quantitative analysis revealed that the flavonoid content of these clones is 60.59 mg/g, 49.62 mg/g, and 41.12 mg/g, respectively. By combining phenotypic and physiological characteristics analysis, we investigated the molecular mechanisms governing differential flavonoid accumulation in the leaves. Results indicated that high-flavonoid lines exhibited enhanced antioxidant capacity, increased metabolic investment during synthesis, improved flavonoid transport efficiency, and stronger stress tolerance. The expression levels of some genes belonging to FLS family showed positive correlations with flavonoid content, while different members of the 4CL gene family showed varying correlation patterns. Furthermore, the expression levels of C12RT1 (EVM0013999), FLS (EVM0004800),4CL (EVM0019469/11395), and FG3 (EVM0020415/13578) were positively correlated with flavonoid content, whereas the expression levels of FG2 (EVM0002360) and LAR (EVM0004274) were negatively correlated with flavonoid content.

**Discussion:**

Our study proposed three distinct flavonoid synthesis strategies. Plants rich in flavonoids may enhance their synthesis capacity by upregulating the expression of key genes such as 4CL and FLS, while downregulating gene expression in competitive pathways.

## 1 Introduction

*Xanthoceras sorbifolia* (*X. sorbifolia*), a deciduous tree or shrub endemic to China belonging to the Sapindaceae family, exhibits remarkable cold, drought, and salt-alkali resistance, making it an important economic species in arid and semi-arid regions (An et al., 2016). The seeds of *X. sorbifolia* contain approximately 40–50% oil, predominantly long-chain unsaturated fatty acids, suitable for biodiesel production. Notably, these seeds can yield about 3% nervonic acid at 70 °C—a monounsaturated long-chain fatty acid with neurotrophic properties, rendering *X. sorbifolia* oil a valuable health supplement (Gao et al., 2024). Widely recognized as a woody oilseed tree with ornamental value, *X. sorbifolia* produces abundant white blossoms during flowering, with petal centers undergoing color changes, creating a striking visual effect. Recent studies have revealed that its leaves are rich in flavonoids and saponins, demonstrating significant medicinal potential (Li et al., 2025). Consequently, developing the leaf utilization of this species represents a promising research avenue with substantial commercial value.

Flavonoids represent a major class of polyphenolic secondary metabolites ubiquitously distributed in plants, with their basic structure consisting of two benzene rings (A and B) connected by an oxygen-containing heterocycle (C), forming a C6-C3-C6 skeletal framework (Dias et al., 2021) This extensive compound family includes subtypes such as anthocyanins, proanthocyanidins (PAs), flavonols, and isoflavones, which not only confer characteristic red, purple, and blue colors to plant organs but also participate in various physiological processes including defense against ultraviolet radiation, pathogen infection, and oxidative stress (Quesada-Vázquez et al., 2024; Li et al., 2025). Modern pharmacological studies have demonstrated that the health benefits of flavonoids primarily stem from their potent antioxidant properties. These compounds effectively neutralize free radicals and reactive oxygen species, mitigating oxidative stress-induced cellular damage. Additionally, they maintain critical physiological functions by regulating key cellular signaling pathways such as NF-κB, MAPK, and PI3K/Akt (Górniak et al., 2018; Intharuksa et al., 2024).

The biosynthesis of flavonoids follows the phenylpropanoid pathway, catalyzed by a series of enzymes encoded by structural genes. Early biosynthesis genes (EBGs) such as *CHS*, *CHI*, and *F3H* play crucial roles in encoding key flavonoid-related enzymes (e.g., chalcone synthase, chalcone isomerase, flavonoid 3-hydroxylase) and regulating the flavonoid biosynthesis pathway, whereas late biosynthesis genes (LBGs) like *DFR*, *ANS/LDOX*, and *UFGT* determine the accumulation of specific branch products (Liu et al., 2021). Extensive research has been conducted on flavonoid synthesis across various plant species. Relevant studies indicate that sweet oranges grafted onto trifoliate orange rootstocks (*Poncirus trifoliate*) exhibited significantly higher total flavonoid content in peels compared to those grafted onto mandarin orange rootstocks (*C. junos Siebold ex Tanaka*) (Li et al., 2023). Furthermore, the rootstock of grape (*Vitis vinifera* L.)can influence the flavonoid metabolism of scions by regulating genes involved in phenylpropanoid metabolic pathways (e.g., *STS*, *ANS*, *FLS*) and the expression of transcription factors such as *MYB* and *WRKY* (Zhang et al., 2022). Additionally, environmental signals affect flavonoid metabolism by modulating the activity of transcription factors. In *Cissus rotundifolia* (a member of the Vitaceae family), drought stress induces upregulation of *FLS* (flavonol synthase) and *LAR* gene expression, promoting the accumulation of flavonol compounds such as myricetin and astragalin, thereby enhancing the plant’s antioxidant defense capacity (Li et al., 2025). In recent years, significant progress has been made in the study of flavonoids in *X. sorbifolia*. Huo et al. conducted a genome-wide association study and flavonoid gene mining (Huo et al., 2025), while Zhang et al. optimized the flavonoid extraction process from *X. sorbifolia* flowers (Zhang et al., 2021). Liu et al. completed telomere-to-telomere (T2T) gapless genome assembly for haplotype analysis of two *X. sorbifolia* varieties (single-flowered variety PBN-43 and double-flowered variety PBN-126) (Liu et al., 2025).

Although omics research on *X. sorbifolia* has achieved considerable progress, studies focusing on flavonoids remain relatively limited. In this study, we aimed to investigate the molecular basis underlying differential flavonoid accumulation among distinct *X. sorbifolia* clones. To this end, we selected three clones with high, medium, and low total flavonoid contents. Through transcriptomic analysis, our objectives were (An et al., 2016): to determine the phenotypic and physiological differences among clones (Gao et al., 2024); to identify differentially expressed flavonoid-related genes among the clones; and (Li et al., 2025) to explore the potential accumulation mechanisms of flavonoids in *X. sorbifolia*.

## 2 Materials and methods

### 2.1 Plant materials and treatments

**Figure 1:**
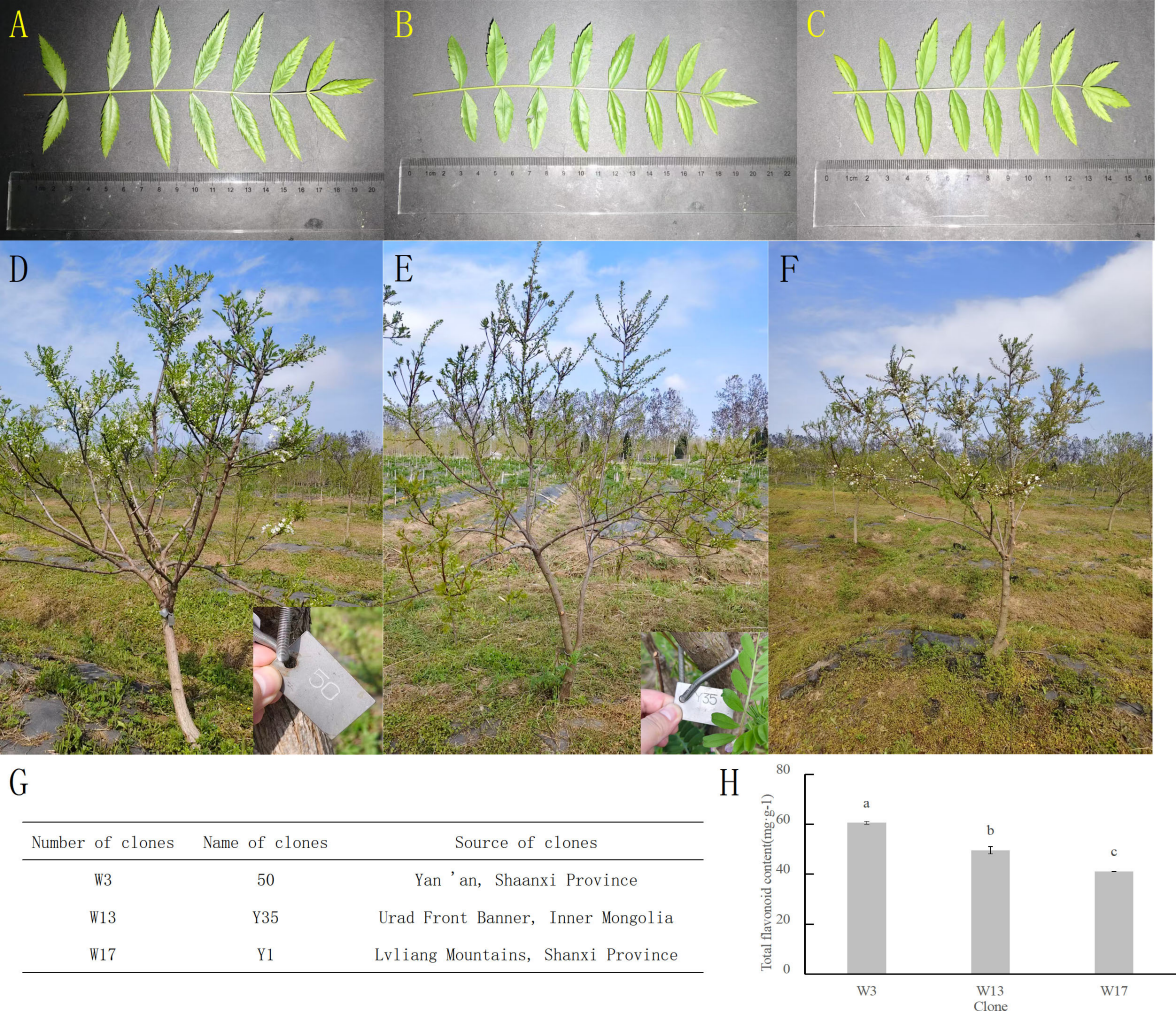
Morphological characteristics of leaves and saplings of tested *X. sorbifolia*, seed source information, and flavonoid content. **(A-C)** Leaf morphotype diagram: **(A)**: W3, **(B)** W13, **(C)** W17. **(D-F)** Sapling morphotype diagram: **(D)** W3, **(E)** W13, **(F)** W17(The name label tag of this clone is missing). **(G)** Scions source information. **(H)** Flavonoid content. Different lowercase letters indicate significant differences among treatments (p < 0.05, Duncan's test).

Four-year-old *X. sorbifolia* grafted saplings were used as experimental materials. Two-year-old saplings were used as rootstocks, with scions selected from healthy one-year-old branches of mature trees. The parent tree was a superior cultivar developed through multi-year selection, aged 10–15 years, 2–3.5 meters in height, with a trunk diameter of 10–20 cm and a crown spread of 2.5–4 meters. Morphological diagrams of leaves and saplings, and specific details are listed in **Figures 1A, B**. The experiment was conducted at the Yancheng Tree Farm, Yancheng City, Jiangsu Province (119°27’–120°54’ E, 32°34’–34°28’ N), characterized by a subtropical monsoon climate with an annual average temperature of 13.9–14.5 °C and annual rainfall of 980–1100 mm. A completely randomized block design was employed, with three plots each containing 10 saplings. Saplings were planted at a density of 3 m × 4 m using raised beds with central drainage ditches 50–60 cm deep. Standard soil, fertilization, and irrigation practices were maintained. Initial laboratory screening identified three *X. sorbifolia* clones (W3, high; W13, medium; W17, low) with statistically significant differences in total flavonoid content (*P* < 0.05) for further sampling (**Figure 1B**).

Sampling occurred in mid-June 2023. For each clone, three healthy, pest-free trees were selected. Twenty mature compound leaves from the upper and middle parts of each plant were collected. Each leaf was placed in a sealed plastic bag; one portion was labeled and stored with ice packs, while the other was transferred to a liquid nitrogen tank. Upon return to the laboratory, ice-pack samples were immediately washed for phenotypic data analysis, while liquid nitrogen samples were stored at -80 °C for subsequent use.

### 2.2 Phenotypic data and physiological parameter determination

#### 2.2.1 Growth and leaves characteristics

Phenotypic data were collected in June 2023. Seedling height, crown width, ground diameter (at ground level), and leaf thickness were measured using a tape measure and digital vernier caliper. Compound leaves were scanned with an Epson scanner, and leaflet area and number per compound leaf were calculated using Adobe Photoshop CS6 software. The fresh weight of 100 leaves was measured with an analytical balance. Leaves were fixed at 105 °C for 15 minutes and then dried at 60 °C to constant weight before measuring dry weight.

#### 2.2.2 Chemical component content

To determine the physiological parameter in *X. sorbifolia*, dried leaves were ground into powder. After removing lipophilic impurities with petroleum ether (30 mL, refluxed at 60 °C for 30 min), ultrasonic-assisted extraction was performed under optimized conditions: 70% ethanol (v/v), feedstock-to-liquid ratio of 1:30 (g/mL), extraction temperature of 60 °C, with three extractions each lasting 20 min. The combined extracts were concentrated under reduced pressure and reconstituted in 70% ethanol to a final volume of 50 mL. Total flavonoid content was determined using the aluminum nitrate colorimetric method (Hao et al., 2013) with rutin as the standard. Total polyphenol content via the Folin phenol method according to national standards (National Administration for Market Regulation and National Standardization Administration, 2018), using gallic acid as the standard. Total saponin content was measured using the vanillin–acetic acid perchloric acid colorimetric method (Qiu et al., 2020) with diosgenin as the standard.

#### 2.2.3 Antioxidant properties analysis

We conducted an antioxidant performance analysis using dried leaves powder of *X. sorbifolia* from Section 2.2.2 as the material. The DPPH free radical scavenging rate was measured as described by Chen Keke and Qiang Yi (Chen and Qiang, 2018), and the ABTS free radical scavenging rate followed Yang Quanli et al (Yang et al., 2022), with suitable modifications.

#### 2.2.4 Key enzymes activities

Similarly, the leaves powder specified in 2.2.2 was used as the material for determining the relevant enzyme activity. The activities of PAL, C4H, and 4CL enzymes in the leaves were measured according to Chen Lei and Fan Bingyou (Fan et al., 2006; Chen et al., 2013), with appropriate modifications.

### 2.3 Transcriptomic analysis

#### 2.3.1 RNA extraction and quality assessment

Total RNA was extracted using the FastPure^®^ Universal Plant Total RNA Isolation Kit (Vazyme, Nanjing, China) according to the manufacturer’s protocol. RNA integrity was assessed by 1.2% agarose gel electrophoresis, and RNA purity and concentration were quantified using a NanoDrop™ 2000 spectrophotometer (Thermo Fisher Scientific, USA). Only RNA samples with A260/A280 ratios between 1.9–2.1, A260/A230 ratios > 2.0, and intact 28S/18S rRNA bands were used for subsequent analysis. Reverse transcription was performed using the HiScript^®^ III RT SuperMix for qPCR (with gDNA wiper) (Vazyme, Nanjing, China).

#### 2.3.2 Library preparation and transcriptome sequencing

Three biological replicates were prepared by rapidly collecting fully expanded leaves from identical anatomical positions (the 5th–7th leaves from the apex) of three clonal individuals (W3, W13, W17) at the same developmental stage. After thorough rinsing with sterile deionized water, samples were immediately transferred to RNase-free cryovials, flash-frozen in liquid nitrogen for 30 min, and transported on dry ice to Shanghai Meiji Biomedical Technology Co., Ltd. (Shanghai, China). RNA sequencing libraries were constructed using the TruSeq™ RNA Sample Preparation Kit (Illumina, USA) following the manufacturer’s instructions. Paired-end sequencing (2 × 150 bp) was performed on the Illumina NovaSeq 6000 platform, generating approximately 6 Gb of clean data per sample (Q30 > 90%).

#### 2.3.3 Quantitative real-time PCR validation

To validate the RNA-seq results, qRT-PCR was performed on selected differentially expressed genes using the Taq Pro Universal SYBR qPCR Master Mix kit (Vazyme, Nanjing, China) on a QuantStudio™ 6 Flex Real-Time PCR System (Applied Biosystems, USA). The Actin gene served as the internal control, with amplification primers designed using Primer 5.0 software and synthesized by Nanjing Bioengineering Co., Ltd. Specific primer sequences are listed in **Table 1**.

**Table 1:** qRT-PCR experimental primer sequence.

Gene ID	Forward primer (5′-3′)	Reverse primer (3′–5′)
Actin	ACGTCACACTGGAGTGATGGTTG	TGGGTTGAGAGGTGCTTCAGTAAG
EVM0019469	AATCGGCATTCAACAATACCAGGTG	AGAAAGGGTTCGCCATCGTAGC
EVM0023285	ACCAACCCAAAACACCACCAAAAC	GCACCAGAGTCGTCACAATCAATG
EVM0015200	GAGGCGTTTCATGGCTTCAACTG	CAGCGAGCGGTGGATCTACTATC
EVM0011987	AAGGTTGTCCGTCTGTCTATGTTCC	AGGTGGCTGAATTTCTCTTTGTTGC
EVM0008511	GTCCTTAAACAGCCAGAACCACAAC	CACCACAACCAAAGTAACTGACCTG
EVM0004274	CAGGCGTTTGATTGAAGAGTTGGG	AGGATGATGGTTGTCGTAGTAAGGC
EVM0013999	GAGACTTGACCAACCAGACAATGC	ACTTGCCACATCCTCAGCTTCC
EVM0013578	TGACGGAGGCTGATCTGTTGAAG	ATTGTCGCTCTGTGAATGGAATGC
EVM0002360	ACAGGAGTGGAGGTGAAGAGGAG	GGAAACACCAGGCTTCTTGTCAAC
EVM0014204	ATACTGGGTCGGTCTGGGAAATC	GTCCTCCTCCGCCGTGAAC
EVM0012697	CACACCCACTTGAAGAAGAGACTG	CTGCGGCGGCGACATTG
EVM0002285	GGAACATGGACACCTGAAGAAGAC	CACCTTGATAGACCTGCATACTTGG
EVM0019008	TTCTCCACCTTACAACAGCAACAAC	GTCTTCTGTCTTCTGGTCCTCCTC
EVM0013316	GCACTACCCACCTACCAGAAACC	CATTGCTTGTTGGTGCGGACTAG
EVM0011442	GCTCTTCATCGAAATCGCCACAG	CGACCTCTGCACCTCCTTCTTG
EVM0013951	TCATCGTCGGATTCTTCGTCTTCAG	TCTCTCTGGTTTCTGGGTTGTTCTC
EVM0002977	ACACCAACTCCCCGACATTGC	CTCCGAACCATCTCCATCACCTG

### 2.4 Data analysis

Excel 2020 was used for data calculation; SPSS 24.0 for correlation analysis, principal component analysis, and cluster analysis; and the Meiji Bio Cloud platform for transcriptome analysis. The software parameters used by this cloud platform are listed in **Supplementary Table S1**.

## 3 Results

### 3.1 Analysis of growth index differences in *X. sorbifolia* clones

**Figure 2:**
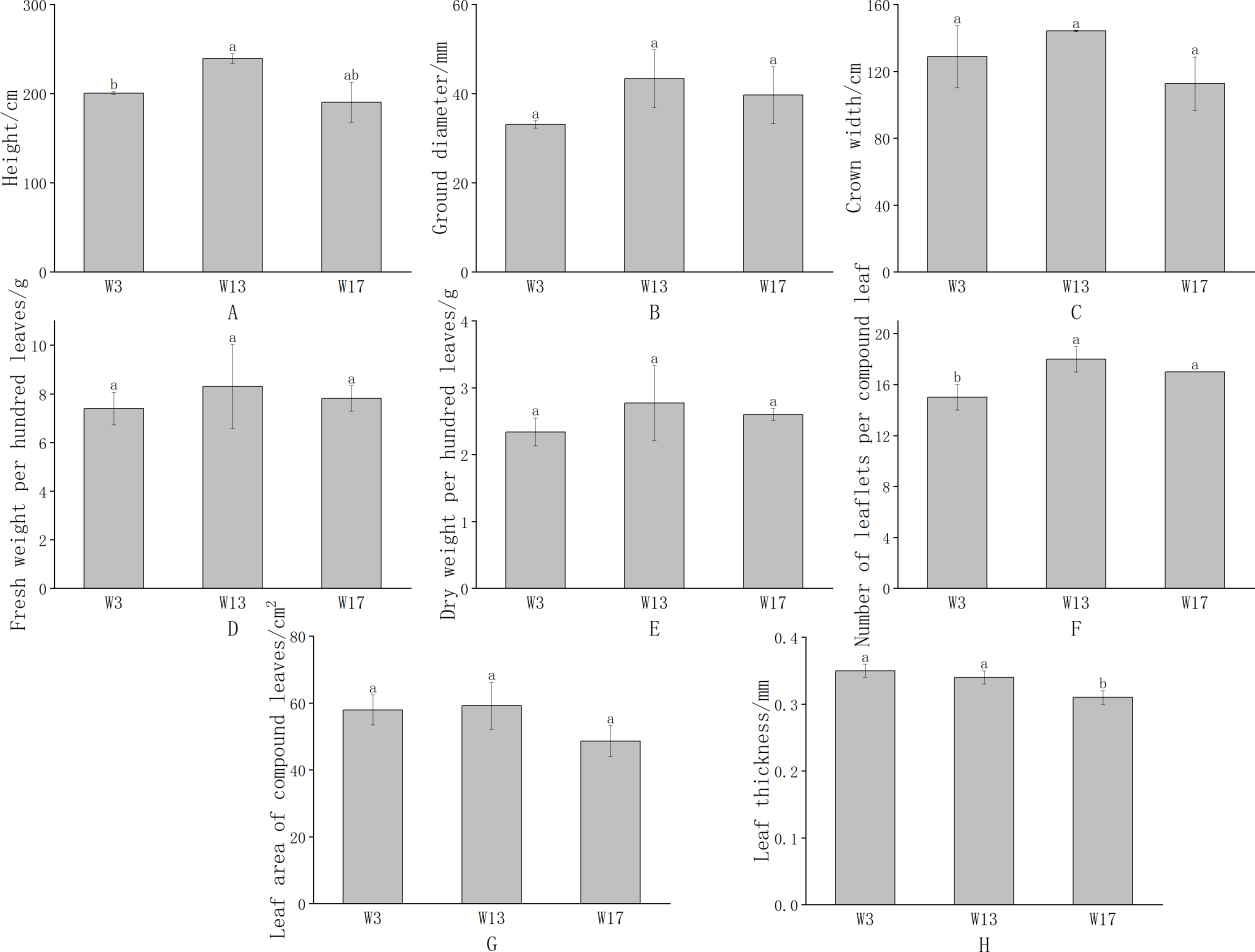
Growth indices of *X. sorbifolia* clones. **(A)** Height. **(B)** Ground diameter. **(C)** Crown width. **(D)** Fresh weight per hundred leaves. **(E)** Dry weight per hundred leaves. **(F)** Number of leaflets per compound leaf. **(G)** Leaf area of compound leaves. **(H)** Leaf thickness. Different lowercase letters indicate significant differences among treatments (p < 0.05, Duncan's test).

Prior to initiating this study, we selected three clones—W3, W13, and W17—as screening criteria based on their flavonoid content, with specific values as follows: W3 (high, 60.59 mg/g), W13 (medium, 49.62 mg/g), and W17 (low, 41.12 mg/g) (**Figure 1B**). Subsequently, we performed the measurement of phenotypic data. Phenotypic data measurement of the three clones revealed that despite significant differences in flavonoid content, no remarkable variations were observed in plant or leaf phenotypes. Among the measured parameters, the clones showed no significant differences in ground diameter, crown width, fresh weight, dry weight, or compound leaf area. The W13 plant exhibited the highest height of 239.5 cm, showing a statistically significant difference compared to W3 and W17. The number of leaflets in the compound leaves of W3 was significantly fewer than those of W13 and W17, totaling 18. Additionally, compared to other clone plants, the leaf thickness of W17 was significantly thinner, measuring 0.31 mm (**Figures 2A–H**).

### 3.2 Physiological index difference analysis of *X. sorbifolia* clones

**Figure 3:**
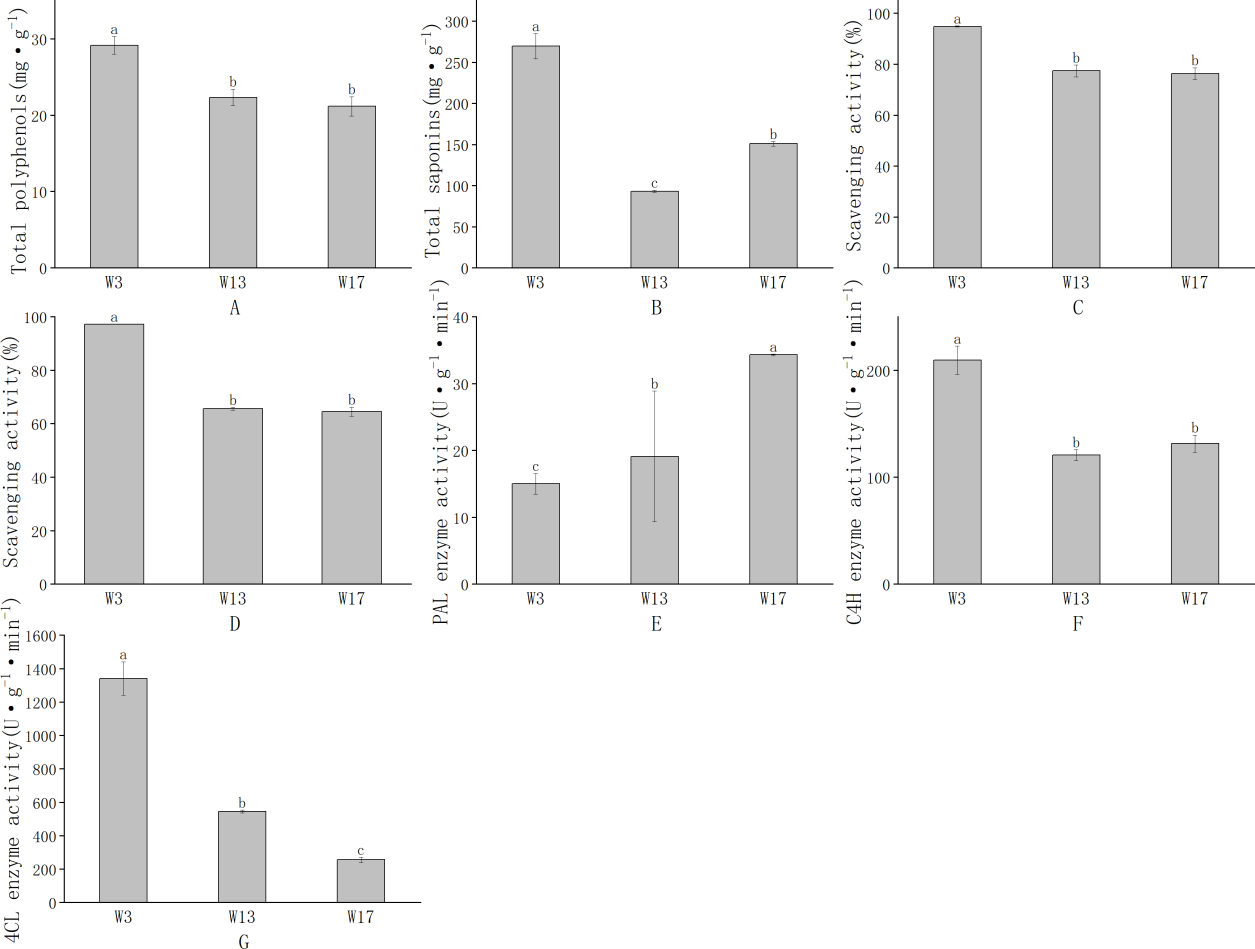
Physiological indices of the tested *X. sorbifolia* clones. **(A)** Total polyphenol content. **(B)** Total saponin content. **(C)** DPPH· scavenging activity. **(D)** ABTS^+^ scavenging activity. **(E)** PAL enzyme activity. **(F)** C4H enzyme activity. **(G)** 4CL enzyme activity. Different lowercase letters indicate significant differences among treatments (p < 0.05, Duncan's test).

To further investigate the differences among the various clones, the researchers measured key physiological indicators. Compared with other strains, W3, which exhibited the highest flavonoid content, demonstrated significantly higher total polyphenol content (29.15 mg/g), total saponin content (269.61 mg/g), DPPH scavenging capacity (94.83%), ABTS^-^ scavenging efficiency (97.24%), C4H enzyme activity (209.34 U·g^-1^·min^-1^), and 4CL enzyme activity (1,340.15 U·g^-1^·min^-1^). Conversely, W17, which had the lowest flavonoid content, showed significantly higher PAL enzyme activity (34.30 U·g^-1^·min^-1^). No strong correlation was observed between total saponin content and flavonoid content: although W3 with the highest flavonoid content had the highest total saponin content, W13 with moderate flavonoid content exhibited the lowest total saponin content at 93.14 mg/g (**Figures 3A–G**).

### 3.3 Transcriptome quality assessment and function annotations

RNA-Seq sequencing using the Illumina NovaSeq 6000 platform generated 9 cDNA libraries. After raw data processing, Clean Reads (**Table 2**) were obtained for subsequent analysis. Post-quality control, average Clean Reads for W3, W13, and W17 were 48,788,492, and 58,506, respectively, with the highest value reaching 50,977,013. Alignment with the *X. sorbifolia* reference genome showed a total alignment rate exceeding 95.74%, unique alignment rates between 86.45% and 87.35%, Q30 base percentage above 94.91%, and GC content between 45.41% and 45.74%. The dataset, characterized by low sequencing errors and high quality, is suitable for further analysis.

**Table 2:** Statistical table of sequencing data.

Sample	Raw reads	Clean reads	Total Mpped	Multiple Mpped	Uniquely Mpped	Q30 (%)	GC content (%)
W3~1	46,657,084	46,404,408	44,538,039(95.98%)	4,178,671(9.0%)	40,359,368(86.97%)	95.33	45.59
W3~2	55,822,816	55,509,046	53,319,740(96.06%)	5,138,777(9.26%)	48,180,963(86.8%)	95.58	45.52
W3~3	44,716,750	44,452,022	42,663,439(95.98%)	3,999,821(9.0%)	38,663,618(86.98%)	95.36	45.54
W13~1	56,978,340	56,601,072	54,343,859(96.01%)	5,330,085(9.42%)	49,013,774(86.6%)	95.42	45.60
W13~2	67,637,498	67,233,194	64,539,487(95.99%)	6,077,038(9.04%)	58,462,449(86.95%)	95.36	45.63
W13~3	51,995,568	51,686,684	49,487,115(95.74%)	4,802,982(9.29%)	44,684,133(86.45%)	95.14	45.57
W17~1	49,812,170	49,525,168	47,739,164(96.39%)	4,480,759(9.05%)	43,258,405(87.35%)	95.23	45.74
W17~2	51,929,706	51,620,848	49,563,325(96.01%)	4,503,712(8.72%)	45,059,613(87.29%)	94.91	45.72
W17~3	52,058,692	51,785,022	49,728,176(96.03%)	4,618,597(8.92%)	45,109,579(87.11%)	95.19	45.41

W3 (1~3), W13 (1~3), and W17 (1~3) represent three biological replicates for clones W3, W13, and W17, respectively.

**Table 2** Statistical table of sequencing data: W3 (1~3), W13 (1~3), and W17 (1~3) represent three biological replicates for clones W3, W13, and W17, respectively.

Principal component analysis (PCA) based on expression levels revealed clear distinctions among the three clones and distinct clusters within each group, indicating good biological reproducibility (**Figure 1C**). Sequencing generated 24,660 expressed genes. Annotation using six major functional databases (NR, COG, GO, Pfam, Swiss-Prot, KEGG) identified 23,654 functionally annotated genes, accounting for 95.92% of the total (**Figure 1D**). The distribution was: NR: 23,628 (95.82%), COG: 21,022 (85.25%), GO: 20,461 (82.97%), Pfam: 20,341 (82.49%), Swiss-Prot: 19,503 (79.09%), KEGG: 10,062 (40.80%). Additionally, 8,696 genes were annotated in all six databases, representing 35.26% of the total (**Figure 1D**).

### 3.4 Gene differential expression analysis

**Figure 4:**
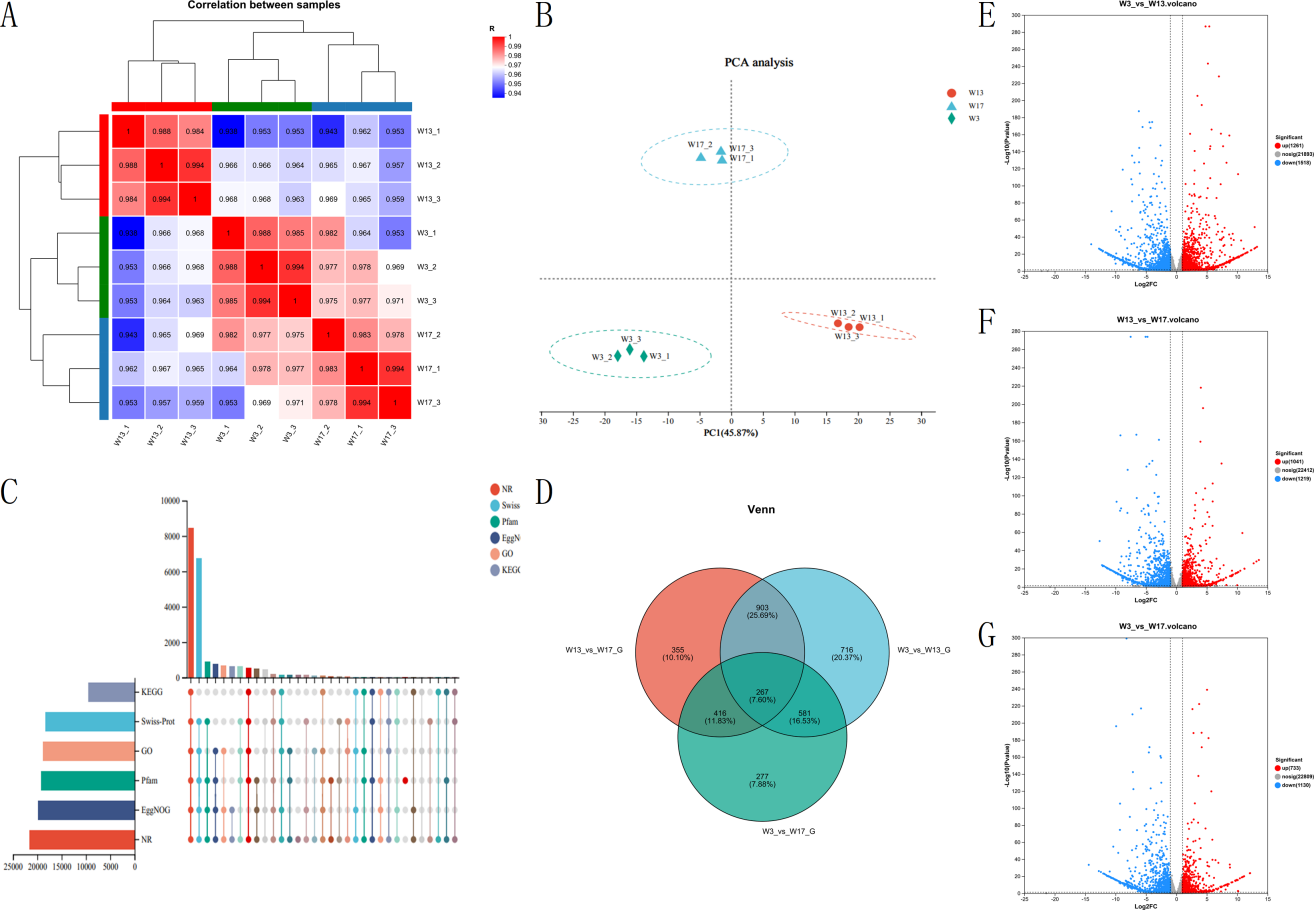
Sample quality assessment and database annotation results of tested *X. sorbifolia*, and differential gene expression in cloned leaves. **(A)** Heatmap of inter-sample correlation analysis. **(B)** Principal component analysis between samples. **(C)** Functional annotation of samples across six databases. **(D)** Venn diagram of DEGs. **(E–G)** Volcano plots of differentially expressed metabolites between groups W3_vs_W13, W3_vs_W17, and W13_vs_W17 (*p*-value <0.05 with fold change>1).

Our correlation analysis revealed high intra-group correlations and low inter-group correlations among treatments, indicating significant separation between groups that allowed for differential expression analysis (**Figure 4C**). In the differential expression analysis, 3,515 significantly differentially expressed genes (DEGs) were identified through pairwise comparisons (|log2Fold Change|>1, *P* < 0.05). Among these, 267 genes exhibited significant differences across all pairwise comparisons, accounting for 7.60% of the total DEGs (**Figure 4D**). The volcano plot demonstrated that the W3 and W13 groups shared 2,467 DEGs (1,117 upregulated genes and 1,350 downregulated genes), while the W3 and W17 groups shared 1,541 DEGs (593 upregulated genes and 948 downregulated genes). The W13 and W17 groups collectively exhibited 1,941 DEGs (889 upregulated genes and 1,052 downregulated genes) (**Figures 4E–G**).

### 3.5 GO and KEGG functional annotation and enrichment analysis

**Figure 5:**
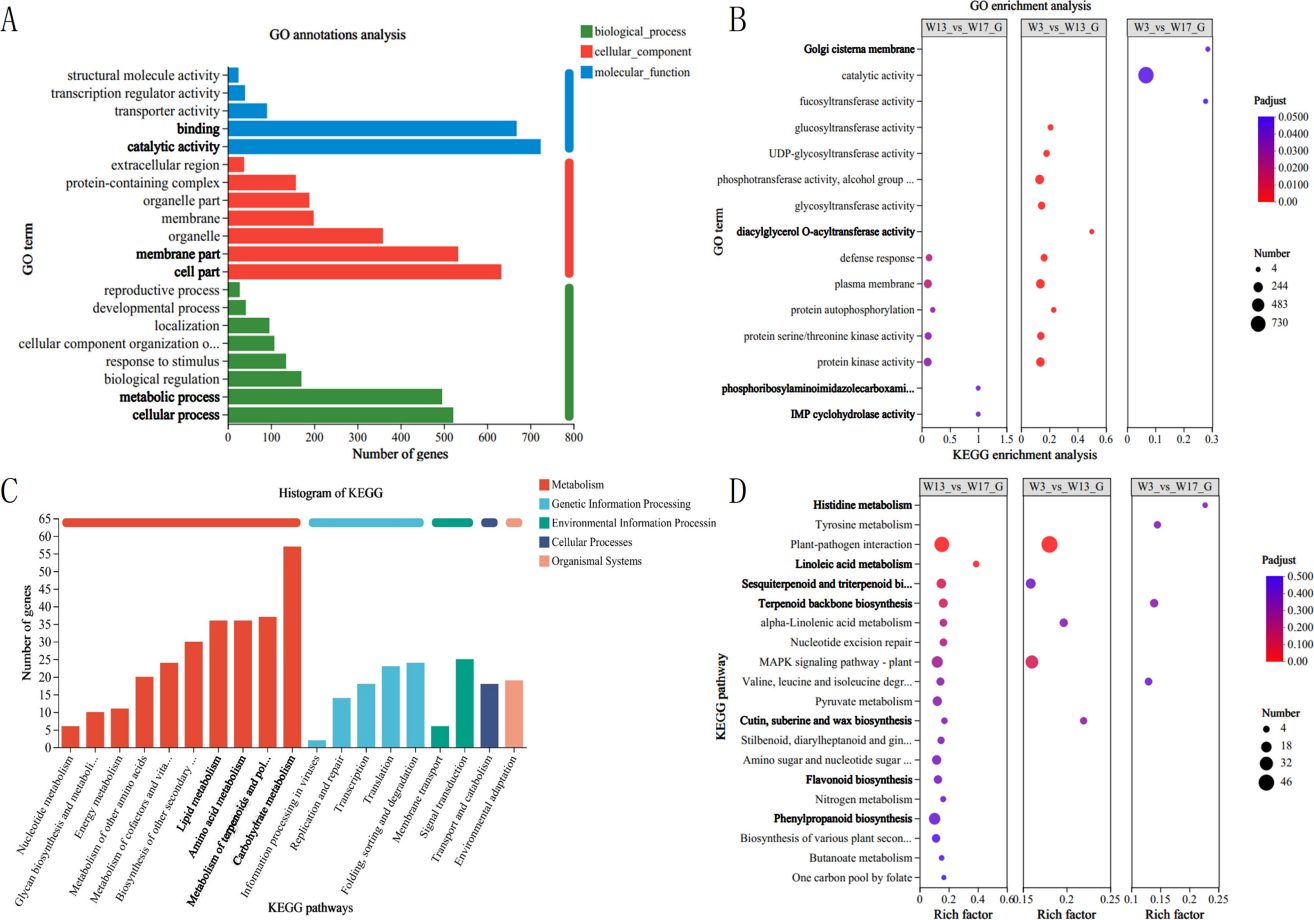
GO and KEGG functional annotation and enrichment analysis of differentially expressed genes. **(A)** GO functional annotation of leaf DEGs. **(B)** GO enrichment analysis of leaf DEGs. **(C)** KEGG functional annotation of leaf DEGs. **(D)** KEGG enrichment analysis of leaf DEGs.

GO annotation of all DEGs identified 43 metabolic processes: 18 in biological processes (1,652 DEGs), 13 in cellular components (2,147 DEGs), and 12 in molecular functions (1,615 DEGs). Among the top 20 entries by abundance, catalytic activity and binding were most frequent in molecular functions (724 and 667 DEGs, respectively). In cellular components, cell parts and membrane parts showed the highest annotation frequency (633 and 533 DEGs). Cellular processes and metabolic processes were the most annotated biological processes (521 and 496 DEGs, respectively) (**Figure 5A**).

GO enrichment analysis (P-adjust ≤ 0.05) on DEGs from the three comparison groups identified the top 10 overlapping enriched pathways (**Figure 5B**). The most significantly enriched GO entries between W13 and W17 were IMP cyclohydrolase activity and phosphoribosylaminoimidazolecarboxamide formyltransferase activity (each with 4 DEGs, Rich factor 1). Diacylglycerol O-acyltransferase activity was most significantly enriched between W3 and W13 (9 DEGs, Rich factor 0.5). The Golgi cisterna membrane pathway was most significantly enriched between W3 and W17 (10 DEGs, Rich factor 0.29). No shared GO entries were found among the three groups.

KEGG annotation of DEGs identified 5 main branches encompassing 19 specific metabolic pathways (**Figure 5C**). These included 10 pathways in Metabolism (267 DEGs), 5 in Genetic Information Processing (81 DEGs), 2 in Environmental Information Processing (31 DEGs), 1 in Cellular Processes (18 DEGs), and 1 in Organismal Systems (19 DEGs). The four metabolic pathways with the highest DEG annotations were carbohydrate metabolism (57 DEGs), terpenoid and polyketide metabolism (37 DEGs), amino acid metabolism (36 DEGs), and lipid metabolism (36 DEGs).

KEGG enrichment analysis (P-adjust ≤ 0.5) identified the top 20 shared enriched pathways (**Figure 5D**). Specifically: W13 and W17 shared 18 metabolic pathways, with linoleic acid metabolism showing the highest enrichment (7 DEGs, Rich factor 0.39). W3 and W13 shared 5 pathways, with cutin, suberine and wax biosynthesis being the most enriched (9 DEGs, Rich factor 0.22). W3 and W17 shared 4 pathways, with histidine metabolism being the most enriched (5 DEGs, Rich factor 0.23). Secondary metabolite pathways among the top 20 included sesquiterpenoid and triterpenoid biosynthesis, terpenoid backbone biosynthesis, flavonoid biosynthesis, and phenylpropanoid biosynthesis.

### 3.6 Gene expression differences of flavonoid biosynthesis-related enzymes

**Figure 6:**
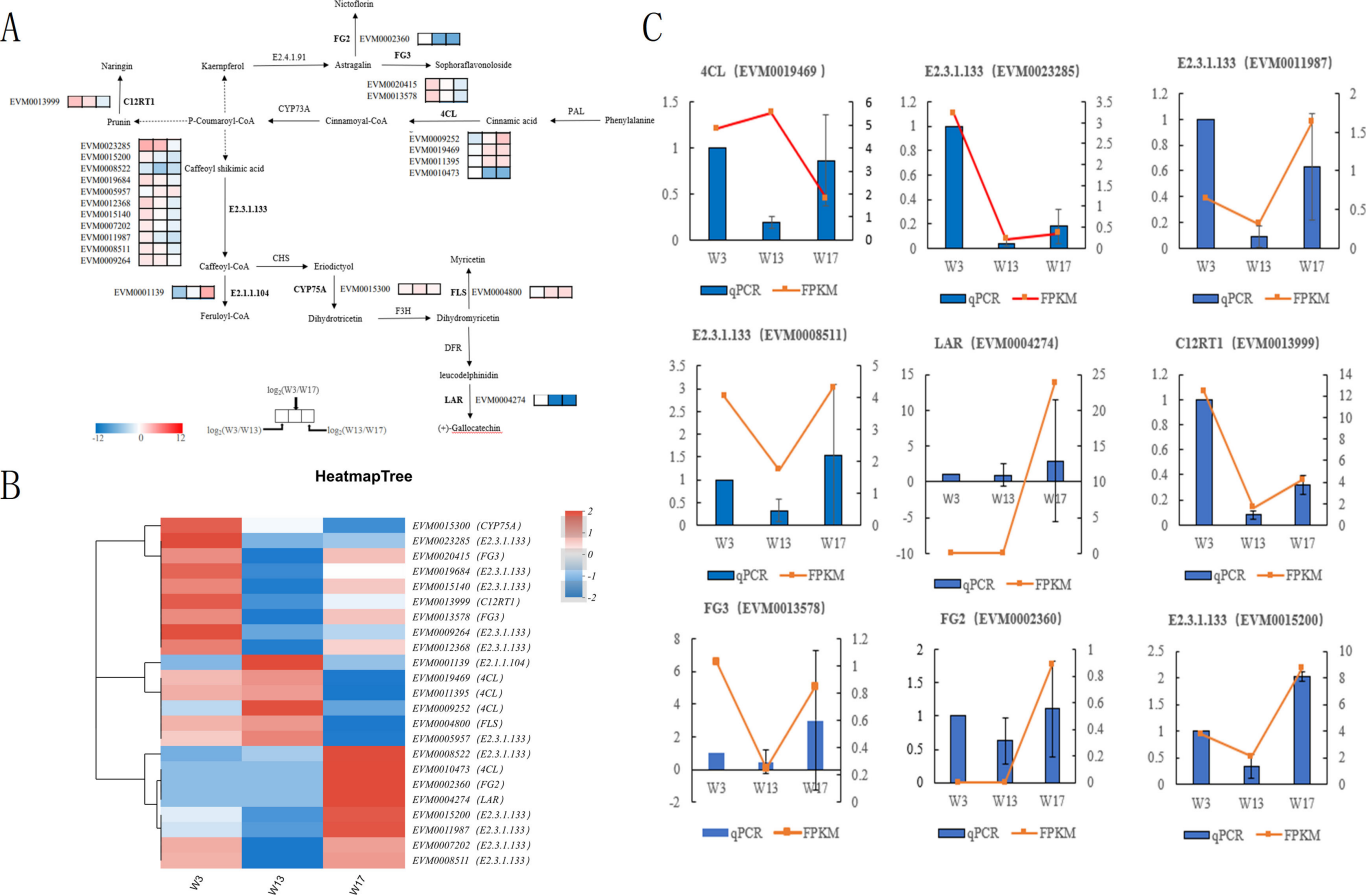
Differential expression of flavonoid biosynthesis pathway enzyme genes, expression heat maps, and qRT-PCR validation results of randomly selected DEGs. **(A)** Differential expression of flavonoid biosynthesis enzyme genes. **(B)** Heat maps of differential gene expression levels. **(C)** qRT-PCR verification of randomly selected DEGs.

To screen for differentially expressed genes related to flavonoids, we conducted a WGCNA analysis correlating with flavonoid content, which largely demonstrated strong correlations with flavonoid levels (**Supplementary Figure S2** ). Subsequently, we performed KEGG enrichment analysis and integrated metabolic pathways for phenylpropanoid synthesis, flavonoid synthesis, and flavonol/flavone synthesis based on the results. In the metabolic pathway map, we observed no significant differences in expression levels of enzyme genes such as *PAL*, *CYP73A*, *CHS*, *F3H*, *DFR*, and *E2.4.1.91* across the three clones. Notably, in W3, the expression levels of *E2.3.1.133* (cassia bark O-hydroxycassia O-glycosyltransferase, *EVM0023285*, *EVM0019684*, *EVM0008511*, *EVM0009264*), *CYP75A* (flavone 3 ‘,5’ -hydroxylase, *EVM0015300*), and *C12RT1* (flavanone 7-O-glucoside 2”-O-β-L-rhamnosyltransferase, *EVM0013999*) were significantly higher than those in other clones. *4CL* (*EVM0019469*, *EVM0011395*) and *FLS* (*EVM0004800*) showed no significant differences with W13 but were significantly higher than W17. *E2.3.1.133* (*EVM0012368*, *EVM0015140*, *EVM0007202*), *FG3* (flavonol-3-O-glucoside/galactosyltransferase, *EVM0020415*, *EVM0013578*), and W17 showed no significant differences, but were significantly higher than W13. *E2.3.1.133* (*EVM0012368*) was significantly lower than the other clones (**Figure 6A**). The expression level differences of the asexual line-specific genes were plotted as a heat map (**Figure 6B**), and their FPKM values are listed in **Supplementary Table S3**.

In W13, the highest expression levels were observed for *4CL* (*EVM0009252*), *E2.1.1.104* (Caffeoyl-CoA O-methyltransferase, *EVM0001139*), and E2.3.1.133 (*EVM0005957*). The expression levels of *4CL* (*EVM0019469*, *EVM0011395*) and *FLS* (*EVM0004800*) were significantly higher than those in W17 but showed no significant difference compared to W3. Similarly, *4CL* (*EVM0010473*) exhibited significantly higher expression levels than W3 but no significant difference compared to W17. Notably, the expression levels of *C12RT1* (*EVM0013999*) and *E2.3.1.133* (*EVM0015200*, *EVM0011987*, *EVM0023285*, *EVM0019684*, *EVM0008511*) in W13 were significantly lower than those in other clones.

In addition to the aforementioned results, we observed that the expression levels of W17’s *E2.3.1.133* (*EVM0008522*, *EVM0015200*, *EVM0011987*), *FG2* (flavonol-3-O-glucoside L-rhamnosyltransferase, *EVM0002360*), and *LAR* (white flower anthocyanin reductase, *EVM0004274*) were significantly highest, while those of *4CL* (*EVM0009252*), *CYP75A* (*EVM0015300*), and *E2.3.1.133* (*EVM0005957*) were significantly lowest.

Our metabolic pathway describes the upstream reactions of flavonoid synthesis catalyzed by 4CL enzymes, where p-coumaroyl-CoA is synthesized to direct the synthesis of various flavonoids. FG2 and FG3 convert astragalosides into nictoflorin and sophoraflavonoloside, respectively, while C12RT1 mediates the synthesis of naringin by Prunin, and p-coumaroyl-CoA serves as a precursor for the synthesis of (+)-gallocatechin and myricetin. Comparative analysis of gene expression levels across different clones revealed that *C12RT1* (*EVM0013999*), *FLS* (*EVM0004800*), *4CL* (*EVM0019469*, *EVM0011395*), and *FG3* (*EVM00020415*, *EVM00013578*) exhibited positive correlations between expression levels and flavonoid content, whereas *FG2* (*EVM0002360*) and *LAR* (*EVM0004274*) showed negative correlations. These differential expression patterns may influence the synthesis of distinct flavonoids, thereby further affecting variations in flavonoid content among clones.

Nine differentially expressed genes (DEGs) were randomly selected from the flavonoid biosynthesis pathway for qRT-PCR analysis. The relative expression level changes of these nine structural enzyme genes were consistent with the transcriptomic data, validating the sequencing accuracy (**Figure 6C**).

## 4 Discussion

### 4.1 Morphological and physiological differences among clonal lines of *X. sorbifolia*

The leaf shape structure serves as the foundation for leaf function, and the structural characteristics of leaves effectively reflect a plant’s growth status and environmental adaptability (Zhou et al., 2024). Currently, there are few studies that establish correlations between leaf morphology and flavonoid compounds. Most studies focus solely on the effects of other factors on leaf morphological traits or flavonoid accumulation. Liu et al. found that light significantly influences leaf morphology and flavonoid accumulation, but no clear correlation was observed between the two (Liu et al., 2016). Our research findings indicate that in the asexual propagation line *X. sorbifolia*, there is only a weak correlation between tree height, ground diameter, leaf morphology, and flavonoid content, which supports the aforementioned study.

Notably, although the morphological characteristics of the various clone lines showed weak correlation with flavonoid content, the activities of PAL, C4H, and 4CL exhibited a significant correlation with flavonoid accumulation. This indicates that metabolic regulation, rather than structural characteristics, is the primary factor governing the chemical differences among these asexual propagation lines. Consistent with our findings, previous studies in *Cinnamomum longepaniculatum* varieties also demonstrated that leaf morphological traits show limited correlation with flavonoid content, whereas enzyme activities in the phenylpropanoid pathway are more predictive of secondary metabolite accumulation (Zhao et al., 2023). Similarly, transcriptome-wide association analysis in hawk tea (*Litsea coreana* var. *sinensis*) revealed that flavonoid accumulation is predominantly associated with genetic variability in metabolic genes rather than leaf phenotypic traits, with structural genes in the phenylpropanoid pathway exerting pivotal influences on flavonoid biosynthesis (Yang et al., 2024). Flavonoid biosynthesis originates from the phenylalanine metabolic pathway, where PAL serves as the first and most critical rate-limiting enzyme, converting phenylalanine into cinnamic acid—the substrate for subsequent flavonoid synthesis (Mao et al., 2025). However, the phenylpropanoid metabolic pathway, located upstream of various plant secondary metabolites, is not only involved in flavonoid synthesis but also participates in lignin and coumarin synthesis (Xue et al., 2023; Li et al., 2024). For example, Yao et al. found that flavonoid DAMs downregulated by GA3 treatment were predominantly enriched in the phenylpropane synthesis pathway, while among the phenolic DAMs upregulated by GA3 treatment, four (36.3%) were lignin biosynthetic metabolites. This suggests a potential metabolic flux diversion between phenylpropane and lignin synthesis pathways in maize, resulting in a negative correlation between flavonoid and lignin synthesis (Yao et al., 2024). Our findings support this hypothesis, indicating that phenylpropane metabolites in *X. sorbifolia* may primarily direct other metabolic pathways (e.g., lignin synthesis), though further experimental validation is warranted. It should also be noted that C4H and 4CL are key enzymes mediating the mid-to-lower pathway reactions of the phenylalanine pathway, and their activity levels to some extent reflect the content of flavonoids (Mao et al., 2025). Our results demonstrate that the activity of both enzymes exhibits a positive correlation with the flavonoid content in clones, which is consistent with expectations. Dong et al. found that benzothiazole (BTH) treatment of Rosa roxburghii Fruit could further enhance the content of secondary metabolites such as lignin, total phenols, and flavonoids by increasing the expression of key genes in the phenylalanine synthesis secondary metabolic pathway and elevating the activity of PAL, C4H, and 4CL enzymes (Dong et al., 2021). This finding corroborates our experimental results.

### 4.2 Correlation between chemical composition and antioxidant activity among clones of *X. sorbifolia*

Polyphenols are important secondary metabolites in plants, primarily functioning by binding to insect digestive enzymes and inhibiting their activity, thereby protecting plants from insect damage (Yao et al., 2026). Saponins, secondary metabolites composed of lipophilic aglycones and hydrophilic glycosides, can be classified into triterpenoid saponins and steroidal saponins. They may alleviate environmental stress by scavenging reactive oxygen species (ROS) (Henry, 2005; Qian et al., 2024). Our experimental results demonstrated a positive correlation between the total polyphenol content and flavonoid content in the three clones; however, interestingly, W13, which exhibited moderate flavonoid content, showed the lowest total saponin content, indicating that saponins in *X. sorbifolia* do not exhibit a strong correlation with flavonoids or polyphenols. This may be attributed to the fact that polyphenols and flavonoids in plants are primarily involved in mitigating abiotic stress, whereas saponins are mainly associated with disease resistance (Byrne et al., 2017; Wu et al., 2023). For instance, in *Astragalus mongholicus*, cutting-induced stress simultaneously upregulates both flavonoid and saponin biosynthesis, but through distinct molecular mechanisms, with saponins primarily serving anti-pathogen functions (Guo et al., 2024). Additionally, flavonoids and polyphenols are synthesized mainly through the phenylpropanoid pathway, while saponins belong to terpenoids and are synthesized via the methylmalonic acid pathway (MVA pathway) or the methylerythritol phosphate pathway (MEP pathway). Although their biosynthetic pathways differ, both require primary metabolites (e.g., acetyl-CoA, pyruvate) as precursor substrates (Luo et al., 2010; Singh et al., 2021; Feng et al., 2024). Therefore, *X. sorbifolia* clones with high flavonoid content likely allocates more of these precursor substrates to flavonoid synthesis.

The DPPH scavenging rate and ABTS²^+^ scavenging rate are primarily used to evaluate the antioxidant capacity of plants. Our results indicate that the antioxidant capacity of *X. sorbifolia* clones exhibits a significant correlation with flavonoid content. The W3 strain not only possesses high flavonoid/polysaccharide content but also demonstrates superior antioxidant performance. As potent natural antioxidants, flavonoids and polyphenols are both associated with enhanced antioxidant capacity (Takatsuka et al., 2022), which is corroborated by the high DPPH scavenging rate and ABTS²^+^ scavenging rate observed in W3. Interestingly, while W13 and W17 showed no significant differences in antioxidant capacity or polysaccharide content, their total flavonoid content differed markedly, which may be attributed to the proportion of flavonoids in these clones that are not involved in antioxidant activity.

Flavonoid synthesis initiates in the cytoplasm, but key steps are catalyzed by the endoplasmic reticulum. The synthesized flavonoids must be stored in vacuoles or secreted extracellularly, a process involving numerous membrane transport proteins and vesicular trafficking mechanisms (Zhu et al., 2016; Chen et al., 2023). Notably, significant differences in gene expression profiles were observed between W3 (high flavone content) and W13/W17 (medium/low flavone content) regarding diacylglycerol O-acyltransferase activity and Golgi membrane vesicle pathways. Diacylglycerol O-acyltransferase, a critical enzyme in triglyceride synthesis, shares the precursor malonyl-CoA with flavonoids (Tsao et al., 2025). Studies suggest that inhibition of lipid synthesis redirects carbon flux toward flavonoid metabolism, and the enrichment of this enzyme in high-flavone plants may indicate sophisticated regulatory mechanisms governing lipid and flavone synthesis (Gai et al., 2020). Concurrently, high enrichment in metabolic pathways indicate that W3 may possess enhanced metabolic capacity. Notably, enrichment analysis revealed differences between W13 and W17 in IMP hydrolase activity and phosphoribosylaminoimidazolecarboxamide formyltransferase activity (involved in *de novo* purine synthesis and associated with energy and cofactor supply (Peano et al., 2012; Hesketh et al., 2019), suggesting that energy and substrate availability may be key limiting factors for flavonoid synthesis in low-flavonoid-content strains. This supports the findings of Wang, et al (Wang et al., 2025).

The cuticle, cork layer, and wax are the primary components of plant epidermal cell walls, forming the first physical defense line against water loss, ultraviolet radiation, and pathogen invasion. This metabolic pathway shares precursor compounds with the phenylpropane metabolic pathway. Plants with high flavonoid content can simultaneously and efficiently synthesize waxes, cutin, and flavonoids, indicating significant advantages in their entire phenylpropane metabolic pathway (Cao et al., 2020; Ojeda-Rivera et al., 2023). This suggests that the core difference between high-flavonoid plants and medium-flavonoid plants lies in their more pronounced investment in phenylpropane/fatty acid precursors during physical barrier construction. Jiang et al. also reported that *Akebia trifoliata* fruit regulates phenylpropane metabolism, leading to structural changes in lignin and cutin (Jiang et al., 2023). Additionally, sesquiterpenes, triterpenes, and terpenes are key components in plant defense against pests and pathogens. The activity of these metabolic pathways indicates that high-flavonoid plants may possess stronger environmental adaptability and stress resistance (Tu et al., 2023).

### 4.3 Differential expression patterns of flavonoid synthesis gene families in different clones of *X. sorbifolia*

The study revealed that the basal level of flavonoid synthesis remains stable in asexual propagation lines. However, different members within the same gene family (i.e., distinct gene subtypes) may exhibit complex expression patterns across various comparative analyses (Deng et al., 2025). For instance, members of the *4CL* gene family demonstrate intricate differential expression patterns among different clones: *EVM0009252* exhibits the highest expression level in W13, while *EVM0019469* and *EVM0011395* show significantly higher expression levels in W3 and W13 compared to W17. This selective expression of *4CL* genes may regulate the supply efficiency of p-coumaroyl-CoA, thereby influencing the metabolic flux distribution in downstream flavonoid biosynthetic pathways.

Our results indicate that the three clonal lines employ distinct strategies in flavonoid synthesis. CYP75A, as a key enzyme in the flavonol synthesis pathway, catalyzes the conversion of dihydrokaempferol to dihydromyricetin, and its high expression may promote the accumulation of myricetin flavonoids in W3, which differs from the findings of Zhang et al. (2025). Additionally, the high expression of the *C12RT1* (*EVM0013999*) in W3 suggests an advantage in the synthesis of flavanone glycosides such as naringin, compounds typically associated with stability, storage properties, and biological activity (Xie et al., 2022). Notably, W13 exhibits high expression levels of *E2.1.1.104*, a key enzyme in the lignin-flavonoid metabolic crossover pathway; its high expression may enhance flavonoid production in W13 by inhibiting lignin synthesis, consistent with the findings of Wu et al (Wu et al., 2025). Meanwhile, the expression of *FLS* (*EVM0004800*) in W13 shows no significant difference from W3 but is significantly higher than in W17, a pattern consistent with FLS’s catalytic role in converting dihydroflavanols to flavonols and potentially explaining W13’s elevated flavonol accumulation levels. Interestingly, the asexual line W17 exhibits a completely distinct expression profile compared to both W3 and W13. As a key enzyme in the proanthocyanidin biosynthesis pathway, LAR catalyzes the reduction reaction converting anthocyanins to catechins; its high expression may promote the accumulation of proanthocyanidins in W17 (Liu et al., 2013). Additionally, some members of the *E2.3.1.133* family may participate in the metabolic pathways of lignin or other phenolic compounds (Kangi et al., 2024), while the high expression of *FG2* is closely associated with the synthesis of nictoflorin-3-O-rutinoside.

Based on the research findings, we hypothesized three distinct flavonoid synthesis modes in *X. sorbifolia*. The high expression of *CYP75A*, *C12RT1*, and specific *E2.3.1.133* genes in W3 may facilitate efficient synthesis of flavonols (e.g., paeonol, quercetin) and their glycoside derivatives. This mechanism resembles the *F3 ‘5’ H* (a *CYP75A* subfamily member) overexpression promoting dihydromyricetin synthesis observed by Wu et al. in Nekemias grossedentata (Wu et al., 2025). In contrast, W13 likely directs more metabolic flux toward lignin monomers and caffeoyl derivatives rather than flavonol glycosides. Zhu et al. demonstrated that genes such as *CCoAOMT* (*EC 2.1.1.104*) and *HCT* (*E2.3.1.133*) exhibit co-expression in Herpetospermum pedunculosum, directing metabolic flux into the lignin pathway, which supports our hypothesis (Zhu et al., 2024). W17 may enhance proanthocyanidin (catechin-like compounds) and specific flavonoid glycosides synthesis while restricting myricetinoid flavonoid production. This pattern aligns with the positive correlation between *LAR* expression and proanthocyanidin accumulation in red-stemmed Medicago sativa varieties (Zong et al., 2025).

### 4.4 Development potential and high-value utilization prospects of flavonoid resources from *X. sorbifolia*

As a tree species with multiple benefits including economic, ecological, and ornamental value, *X. sorbifolia* has traditionally been utilized primarily for its timber and seed oil. However, the abundant flavonoid resources contained in its nutritional organs such as flowers and leaves have not been fully exploited (Zhang et al., 2021; Cui et al., 2025). Compared to the long cultivation cycle for timber production (typically 10–15 years), the harvesting cycle for leaves and flowers is merely one year, and continuous annual harvesting can be achieved through proper pruning. The annual yield of bioactive compounds per unit area is significantly higher than that of traditional utilization methods. At the industrial application level, flavonoid extracts derived from *X. sorbifolia* exhibit superior DPPH and ABTS+ radical scavenging capabilities compared to rutin, along with strong inhibitory effects against Escherichia coli, *Staphylococcus aureus*, and Bacillus subtilis (Zhang et al., 2021). These pharmacological properties endow *X. sorbifolia*-derived flavonoids with broad prospects for applications as functional food ingredients, natural antioxidants, and plant-based antimicrobial agents. Particularly against the backdrop of growing global concerns regarding the safety of synthetic antioxidants (e.g., BHA, BHT), these natural flavonoids represent an ideal alternative with substantial economic potential.

The efficient accumulation of flavonoids in leaves of *X. sorbifolia* can be systematically optimized through genetic improvement, environmental regulation, and cultivation management. Based on our research findings, the following feasible measures can enhance flavonoid content in the leaves of *X. sorbifolia* (An et al., 2016): Variety breeding and asexual propagation. The three tested clones exhibited significant differences in flavonoid levels. Future studies could utilize molecular marker-assisted selection (MAS) technology to identify high-content genotypes and achieve trait stabilization through tissue culture or cuttings propagation (Gao et al., 2024). Nutrient management and precision fertilization. Our research revealed variations in utilization efficiency of flavonoid synthesis substrates and energy among clones. Nitrogen supply levels significantly regulate secondary metabolism: low nitrogen stress typically promotes synthesis of carbon-based secondary metabolites (including flavonoids), while high nitrogen levels favor vegetative growth but dilute active compound concentrations (Huang et al., 2025). Applying low-concentration nitrogen fertilizer during vegetative growth (spring) to facilitate canopy development, followed by nitrogen cessation 4–6 weeks before leaf harvest and increased phosphorus/potassium application to enhance flavonoid conversion and accumulation, proves effective (Li et al., 2025). Stress induction. Studies indicate that appropriate stress application stimulates synthesis of stress-resistant flavonoids (Akhtar et al., 2026). As a drought-resistant tree species, *X. sorbifolia* can benefit from mild drought stress to increase flavonoid content.

## 5 Conclusions

This study systematically compared the growth characteristics, leaf morphology, antioxidant capacity, and flavonoid synthesis-related gene expression profiles of three different flavonoid-content clones of *X. sorbifolia* (high-flavonoid: W3, medium-flavonoid: W13, low-flavonoid: W17), revealing the metabolic characteristics and synthesis mechanisms of high-flavonoid-content *X. sorbifolia*.

The research results indicate that although significant differences in flavonoid content were observed among the various clones, these variations did not substantially affect growth indicators such as tree height, ground diameter, or leaf morphology, demonstrating that high flavonoid accumulation can coexist with favorable growth performance. The high-flavonoid asexual line W3 exhibited enhanced antioxidant capacity, with significantly superior DPPH and ABTS^+^ scavenging rates compared to other lines. At the metabolic level, the high-flavonoid *X. sorbifolia* clone (W3) demonstrated more efficient phenylpropane metabolic pathway activity, with key enzyme activities (e.g., PAL, C4H, and 4CL) showing positive correlations with flavonoid content. Additionally, it exhibited active metabolism in the cuticular layer, waxy substance synthesis, and terpenoid production, suggesting stronger environmental adaptability and stress resistance. Gene expression analysis revealed that the three clones likely employ distinct flavonoid synthesis strategies: W3 promotes efficient synthesis of flavonols and their glycosides through high expression of genes such as *CYP75A* and *C12RT1*; W13 likely directs more metabolic flux toward lignin monomer synthesis; whereas W17 tends to accumulate proanthocyanidins.

In conclusion, through systematic optimization measures such as variety breeding, precision fertilization, and stress induction, the flavonoid accumulation efficiency of *X. sorbifolia* leaves can be further enhanced, thereby promoting its value realization in natural product development and industrial applications.

## Data Availability

The raw data supporting the conclusions of this article will be made available by the authors, without undue reservation. The raw sequencing data reported in this paper have been deposited in the NCBI Sequence Read Archive under accession number “https://www.ncbi.nlm.nih.gov/bioproject/PRJNA1456975” PRJNA1456975. All other data supporting the findings of this study are available within the paper and its supplementary information files.
