# Supplementary figures and images for: Study on differences in flavonoid synthesis in Xanthoceras sorbifolia leaves based on transcriptome analysis
Source: Front Plant Sci. 2026 Jun 19;17:1822700. doi: 10.3389/fpls.2026.1822700 (PMC13328179; doi:10.3389/fpls.2026.1822700)

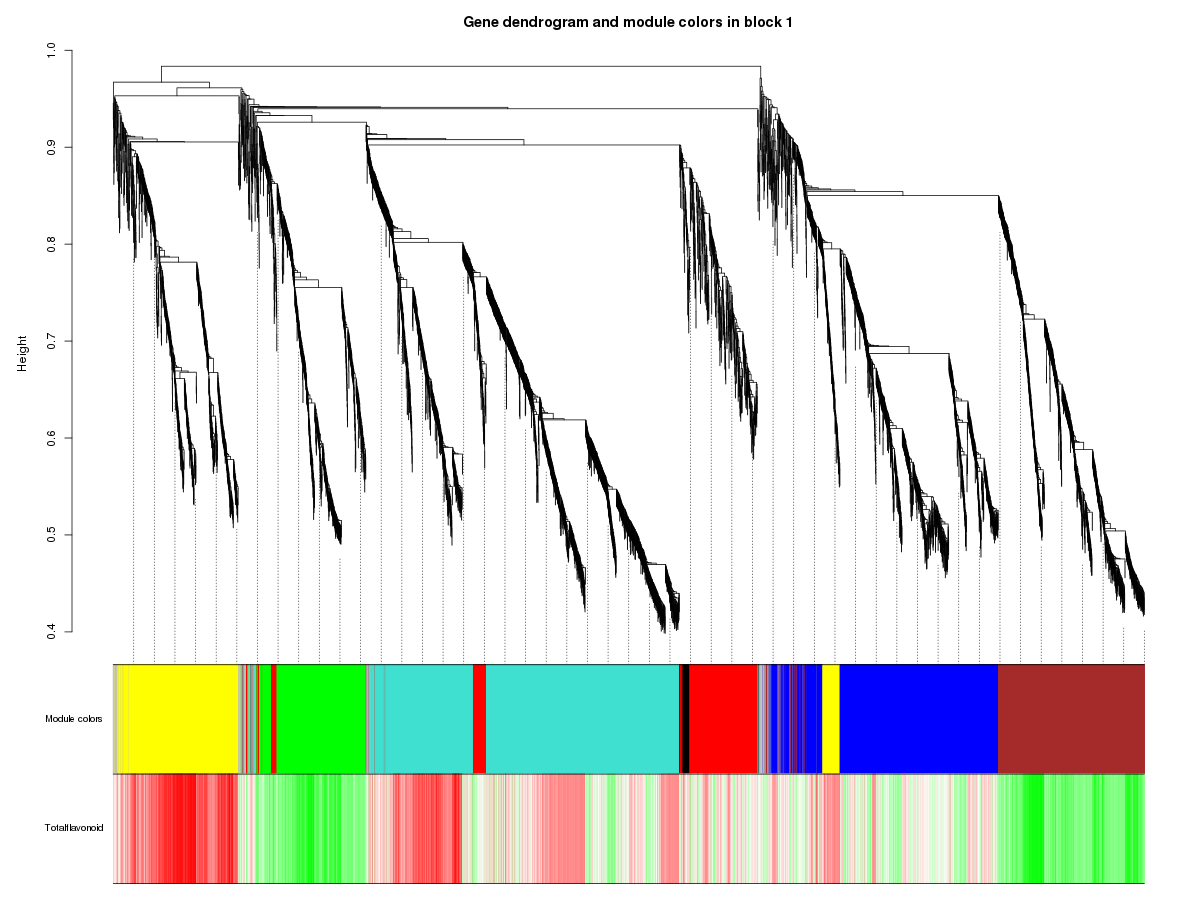

Supplement: Supplementary file 1 [file Image1.png]
